# Fast approximate models of large networks

**DOI:** 10.1186/1471-2202-15-S1-P22

**Published:** 2014-07-21

**Authors:** Bryan P Tripp

**Affiliations:** 1 Centre for Theoretical Neuroscience, University of Waterloo, Waterloo, ON, Canada, N2L 3G1

## 

This work addresses two key challenges in modeling large neural systems such as the primate visual system. The first challenge is that large detailed models require prohibitively expensive computers and long simulation times. The second is that interpretation of simulation results is difficult due to the large number of model parameters. The Neural Engineering Framework (NEF) helps to address the second challenge by defining mappings between neural activity and higher-level behaviour [1,2]. In particular, NEF models behave *approximately* as systems of nonlinear ordinary differential equations in which the number of states is typically about two orders of magnitude less than the number of neurons. The present work consists of a computationally efficient model of this approximation, i.e. of the functional differences between NEF neural-circuit models and corresponding lower-dimensional dynamics. There are only two such functional differences. One is that the neurons behave as if the right-hand sides of the ODEs are somewhat distorted. High-frequency distortions over the state variables impose attractors on feedback networks. There is also compression at large magnitudes of the state variables. The other difference is that spikes introduce broadband noise-like current fluctuations. The present work approximates these differences in a computationally efficient manner. The procedure is to model a group of point neurons and their connections within a network, to approximate their aggregate behaviour as a surrogate “population model”, and then to simulate only the population model.

These surrogate models have two benefits. The first is the practical benefit of a 1-2 orders-of-magnitude reduction in computation. This allows one to run much more complex simulations with the same computer. Efficient parallel implementations are currently in progress, but functionally realistic real-time simulation of 10^7^-10^8^ point neurons is anticipated on a single graphical processing unit (the number depending substantially on network connection statistics). The second benefit is that this model provides a simple and thorough description of the behaviour of complex networks and how it arises from neuron parameters. As an example, we found that fluctuations in post-synaptic current due to synaptic input shift to higher frequencies when the presynaptic spike rates are higher.

The main goal of this work has been to accelerate and enhance insight into NEF models, but the method applies to any network of point neurons with linear synaptic integration. If there are about half as many large singular values in a synaptic weight matrix as there are post-synaptic neurons, the surrogate model will be about as efficient as the full neuron model. However, if there are few large singular values then the population model is much more efficient. Future work will address conductance models of synapses, synaptic plasticity, and further scale improvements.

**Figure 1:**
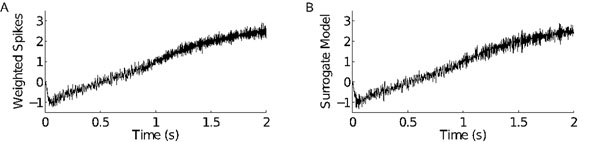
Simulations of 400 neurons that encode a one-dimensional value *x*, which ramps from -1 to 3. **A.** Weighted sum of spikes (weights chosen to optimally approximate *x*), filtered by a synapse model. This approximates synaptic drive from these neurons to another neuron (not simulated). **B.** Efficient surrogate model of the quantity in A.
